# MR-Linac Radiotherapy – The Beam Angle Selection Problem

**DOI:** 10.3389/fonc.2021.717681

**Published:** 2021-10-01

**Authors:** Rik Bijman, Linda Rossi, Tomas Janssen, Peter de Ruiter, Baukelien van Triest, Sebastiaan Breedveld, Jan-Jakob Sonke, Ben Heijmen

**Affiliations:** ^1^ Department of Radiotherapy, Erasmus MC Cancer Institute, Rotterdam, Netherlands; ^2^ Department of Radiation Oncology, The Netherlands Cancer Institute, Amsterdam, Netherlands

**Keywords:** MR-linac, beam angle optimization (BAO), beam angle class solution, automated planning, rectal cancer

## Abstract

**Background:**

With the large-scale introduction of volumetric modulated arc therapy (VMAT), selection of optimal beam angles for coplanar static-beam IMRT has increasingly become obsolete. Due to unavailability of VMAT in current MR-linacs, the problem has re-gained importance. An application for automated IMRT treatment planning with integrated, patient-specific computer-optimization of beam angles (BAO) was used to systematically investigate computer-aided generation of beam angle class solutions (CS) for replacement of computationally expensive patient-specific BAO. Rectal cancer was used as a model case.

**Materials and Methods:**

23 patients treated at a Unity MR-linac were included. BAO_x_ plans (x=7-12 beams) were generated for all patients. Analyses of BAO_12_ plans resulted in CS_x_ class solutions. BAO_x_ plans, CS_x_ plans, and plans with equi-angular setups (EQUI_x_, x=9-56) were mutually compared.

**Results:**

For x>7, plan quality for CS_x_ and BAO_x_ was highly similar, while both were superior to EQUI_x_. E.g. with CS_9_, bowel/bladder D_mean_ reduced by 22% [11%, 38%] compared to EQUI_9_ (p<0.001). For equal plan quality, the number of EQUI beams had to be doubled compared to BAO and CS.

**Conclusions:**

Computer-generated beam angle CS could replace individualized BAO without loss in plan quality, while reducing planning complexity and calculation times, and resulting in a simpler clinical workflow. CS and BAO largely outperformed equi-angular treatment. With the developed CS, time consuming beam angle re-optimization in daily adaptive MR-linac treatment could be avoided. Further systematic research on computerized development of beam angle class solutions for MR-linac treatment planning is warranted.

## Introduction

With the large-scale introduction of volumetric modulated arc therapy (VMAT), the problem of selecting a set of optimal beam directions in treatment planning for coplanar IMRT with static beam configurations has increasingly become obsolete. Current MR-linac (MRL) systems (1–4) do not offer VMAT and only allow coplanar treatment, meaning that the beam angle selection problem for coplanar treatments has re-gained importance. This could also have an impact on daily adaptive re-planning at MRLs in case daily re-optimization of beam angles would result in enhanced daily dose distributions.

Selection of optimal IMRT beam directions with conventional trial-and-error (‘manual’) planning can be extremely challenging and time-consuming. In recent years, many studies have investigated the use of computer optimization of beam angles in non-coplanar IMRT, as often applied in stereotactic (body) radiation therapy (S(B)RT) (5–7). For many treatment sites [e.g., liver (5), lung (6), head-and-neck (8) and prostate (9)], computer optimized beam setups resulted in high-quality plans. Computerized beam angle selection has also been investigated for coplanar IMRT treatments (10–12). Probably related to the introduction of VMAT, there hardly seem to be recent studies. Several treatment planning studies for MRL systems showed adequate and clinically acceptable IMRT plan quality (13–19). All these studies were based on manual beam angle selection, as the treatment planning systems for the available MRLs do not feature computerized beam angle selection.

In a previous study, we developed a workflow for fully-automated, multi-criterial generation of IMRT plans for a high-field MRL (17). For rectal cancer patients, retrospectively generated IMRT plans for clinical beam angles were superior to the clinical IMRT plans, generated with manual planning. The applied optimization workflow also allows integrated optimization of beam angles (BAO) and IMRT profiles. In this study, this BAO feature was explored for rectal cancer, aiming at development of beam angle class solutions to replace time-consuming individualized beam angle selection with minimal plan quality loss. Apart from generation of high-quality initial treatment plans, adequate beam angle class solutions would also be useful for fast daily adaptive re-planning, as this could be limited to re-optimization of intensity profiles. Validation of the plans generated with beam angle class solutions was done by comparison to plans with patient specifically optimized beam angles and plans with equi-angular setups. Often applied equi-angular setups were chosen as a reference to avoid dependence on subjective beam selection by human planners. To provide a strong validation of CS plans (with a maximum of 12 beams), comparative plans with equi-angular setups contained up to 56 beams.

## Materials and Methods

### Patients and Clinical Treatment Planning

**Figure 1:**
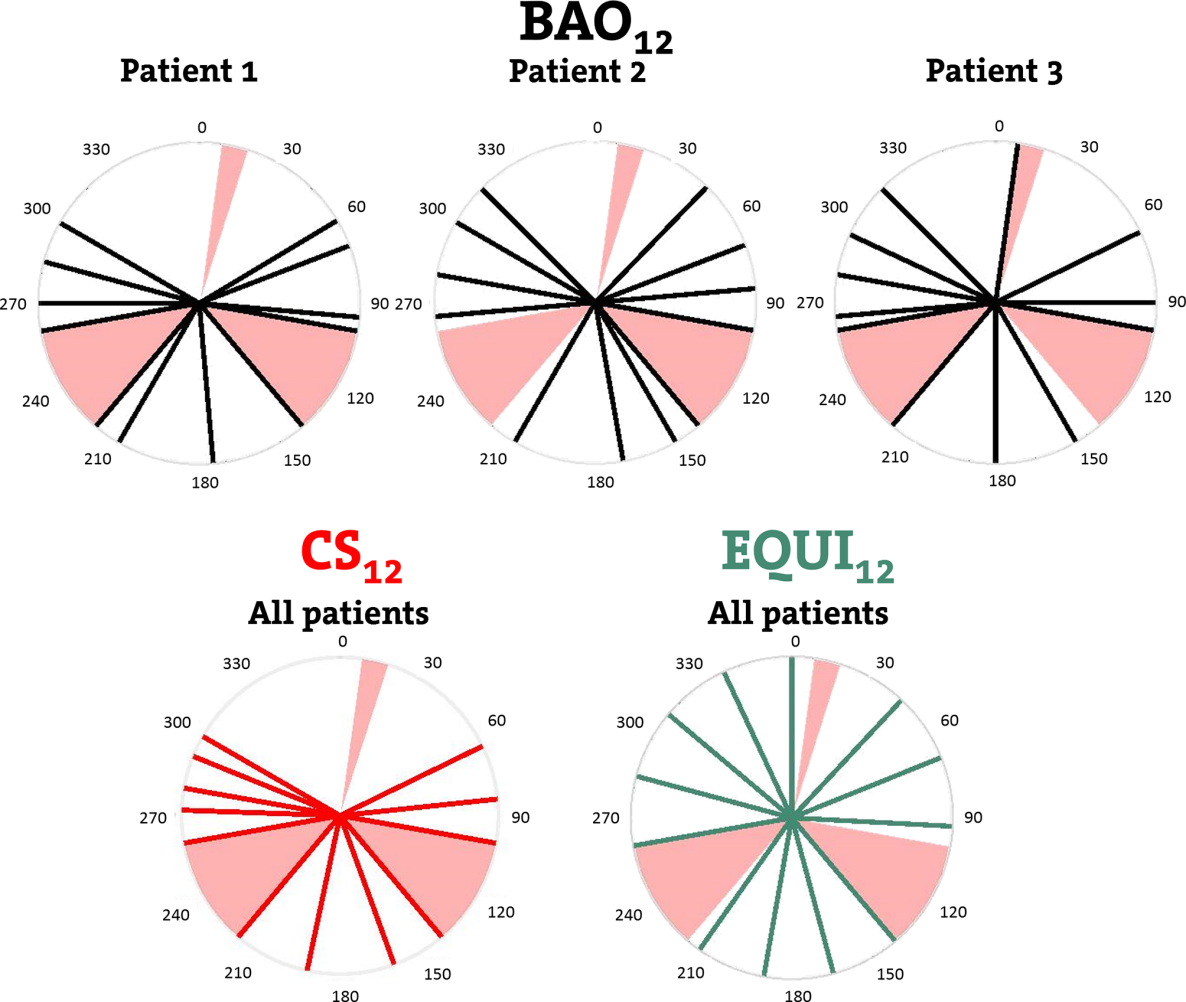
Upper row: individualized beam angles of the 12-beam plans for three example patients, generated with integrated beam profile and angle optimization (BAO). Lower row: beams for CS_12_, the 12-beam class solution, and for EQUI_12_, the 12-beam equi-angular setup. Black, red and green lines represent treatment beams. Beam avoidance areas (BAAs) for the Unity MRL are depicted in pink.

Planning CT-scans of 23 rectal cancer patients, previously treated at the NKI (The Netherlands Cancer Institute, Amsterdam) at a Unity MRL; (Elekta AB, Stockholm, Sweden), were included in this study. The CTV was defined as the combination of GTV, expanded with a 10 mm margin for subclinical disease, and regional lymph node areas (mesorectal, internal iliac, and depending on GTV location and N-stage, obturator and/or presacral). The CTV was expanded with a 10 mm margin in all directions, except for an expansion of up to 15 mm anterior to mesorectal region (20). Around the internal iliac, obturator lymph node areas a margin of 5 mm was taken. All delineated areas were adapted to non-involved structures such as bone structures. The bladder and bowel bag (‘bowel’ in the remainder of this paper) were separately delineated and then joined with exclusion of the overlap with the PTV, to construct a composite OAR (‘OAR’ in the remainder of this paper) that was used for planning. An artificial helper structure in the dorsal part of the patient was used to avoid unacceptable high dose posterior to the PTV, caused by the high magnetic field (17). All patients were clinically treated with the same beam setup, consisting of 9 beams not passing through the three MRL-specific beam avoidance areas (BAAs): the cryostat pipe (gantry angles 8°-18°) and two high attenuation regions of the MRL treatment couch (100°-140° and 220°-260°) (17) (See also pink areas in **Figure 1**). Treatment plans were generated to deliver 50 Gy in 25 fractions, which were considered clinically acceptable in case PTV V_95%_ exceeded 99%, while V_107%_<1-1.5%. Additional planning goals were a maximum reduction of OAR D_Mean_ (first priority), and a PTV D_Mean_ close to the prescribed dose, as well as controlling delivered high and low patient doses (ALARA).

### Automated Plan Generation

All plans in this study were fully automatically generated with the in-house developed Erasmus-iCycle multi-criterial optimizer (Details can be found in (7, 21), and a brief summary is given below), coupled to the same Monte Carlo dose engine as used in the clinical MRL TPS, in order to account for the dosimetric impact of the applied high magnetic field. The system was tuned for generation of high-quality MRL plans for rectal cancer patients, in line with the clinical planning protocol at NKI (17), above). With the dorsal artificial helper structure (*Patients and Clinical Treatment Planning*), potential negative impact of the electron return effect (ERE) on the dose in the patient’s skin was mitigated (17).

Erasmus-iCycle has been developed for fully automated multi-criterial IMRT planning for pre-selected beam angles, or with integrated beam angle optimization. Treatment site specific configurations (‘wish-lists’), consisting of hard constraints and prioritized objectives, are created to ensure that the generated Pareto-optimal plans are also clinically favorable (7, 22, 23). In the plan generation for a patient, the objective functions are minimized sequentially following the order of assigned priorities, while avoiding violations of imposed constraints. After the minimization of a cost function, an extra constraint is added to the optimization problem to ensure that minimization of lower priority cost functions will not result in reduced quality for the higher priorities. In case of integrated beam angle optimization, favorable directions are sequentially added to the plan (7). This approach may for smaller numbers of beams (~7 and lower) in some cases result in a plan quality that is somewhat lower than maximally achievable.

### Construction of Beam Angle Class Solutions (CS) and Validation

In total six CS_x_ with x=7 up to 12 beams were constructed. Construction of each CS_x_ was based on the beam directions found in the BAO_12_ plans of the *N* patients that were used for its creation (in total *M* = *N* ∙ 12 input directions), i.e. also for creation of CS_x<12_, beam directions in BAO_12_ plans were used. The basis for selection of the x directions in CS_x_ was a frequency histogram with the *M* input directions. In a pre-processing step, prior to final CS_x_ beam selection, lower frequencies were added to neighbouring (within 5 degrees) bins with higher frequencies. If pre-processing ended up with more than x beams, a selection was performed among the beams with lowest frequencies, such that remaining beams had a maximum distance to already selected beams with higher frequencies.

For assessing whether the method for CS_x_ construction would generalize to an independent data set, we used the leave-one-out-method, i.e. building 23 models (one per patient), each one constructed with the BAO_12_ plan of *N* = 22 patients (*M* = 22 ∙ 12 input directions), and then compare for the patient that was not involved in CS_x_ construction, the CS_x_ plan with his/her BAO_x_ plan.

After validation of the methodology, final CS_x_ were established using all 23 study patients as input.

### Generated Treatment Plans

For all patients in this study, the following plans were generated and mutually compared. A graphical summary, for x=12 beams, is provided in **Figure 1**.

***BAO_x_***: plans generated with individualized beam profile and beam angle optimization (BAO) for x = 7-12 beams. The candidate beam set consisted of 56 beams distributed over 360°, starting at gantry angle 0°, separated by 5° and excluding the BAAs (**Figure 1**).

***CS_x_***: plans generated with beam profile optimization only, using x = 7-12 fixed beam directions defined by the CS_x_ class solutions (above).

***EQUI_x_***: plans generated with beam profile optimization only, using equi-angular beam setups with x = 9, 12, 15, 19, 24, 29, or 56 beams, while excluding the BAAs.

With the defined BAO_x_, CS_x_ and EQUI_x_, 19 plans were generated for each of the 23 patients (6 BAO_x_, 6 CS_x_ and 7 EQUI_x_ plans), resulting in a total of 437 plans used for plan comparisons.

### BAO, CS and EQUI Plan Evaluations and Comparisons

To avoid bias in OAR plan parameter comparisons, generated plans were rescaled such that 99% of the PTV received 95% of the prescribed dose (conform clinical protocol). Like in clinical practice, the mean dose in the composite OAR was then the most important parameter for comparisons of BAO_x_ with CS_x_ and EQUI_x_, but involved bladder and bowel doses were also evaluated separately using D_Mean_ and V_45Gy_ (24, 25). Furthermore, PTV V_107%_, PTV D_Mean_, conformity index (CI, defined as V_95%_/V_PTV_) and the dose bath (V_10Gy_, V_20Gy,_ V_30Gy,_ V_40Gy_ in the patient) were considered in plan evaluations and comparisons. Two-sided Wilcoxon signed-rank tests were used for statistical analyses, with p-values <0.05 indicating statistical significance of plan parameter differences.

## Results

### Validation of CS_x_ Construction and the Final CS_x_

**Figure 2:**
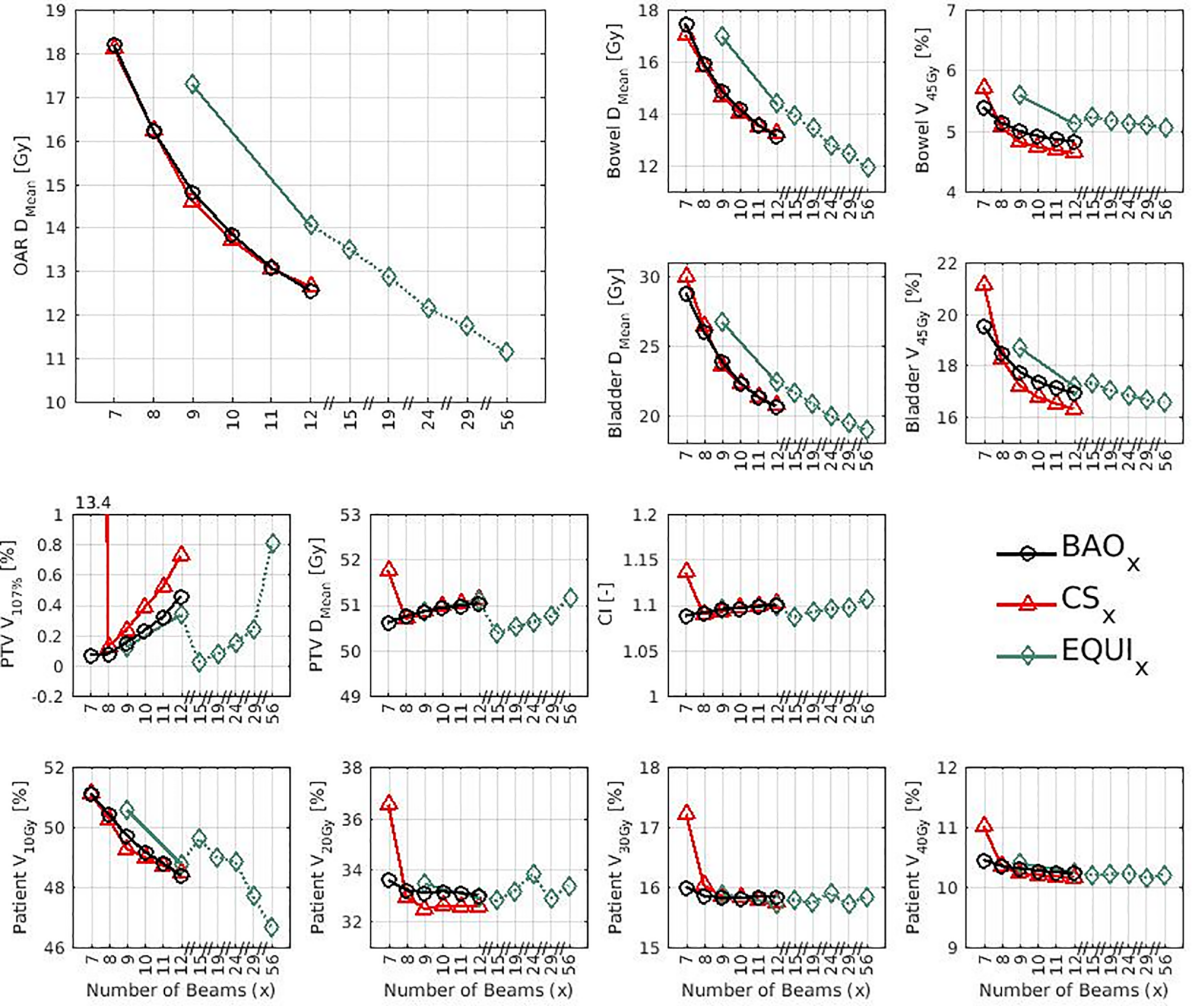
Mean dose parameters for the 23 study patients. The top left shows the results for the composite OAR, the clinically most important healthy tissue structure. Note the non-continuity of the x-axes. P-values for all mutual comparisons of beam angle approaches can be found in **Figures A3**-**A14** in Electronic Supplement A. Leave-one-out data was used for CS_x_.

**Figure 3:**
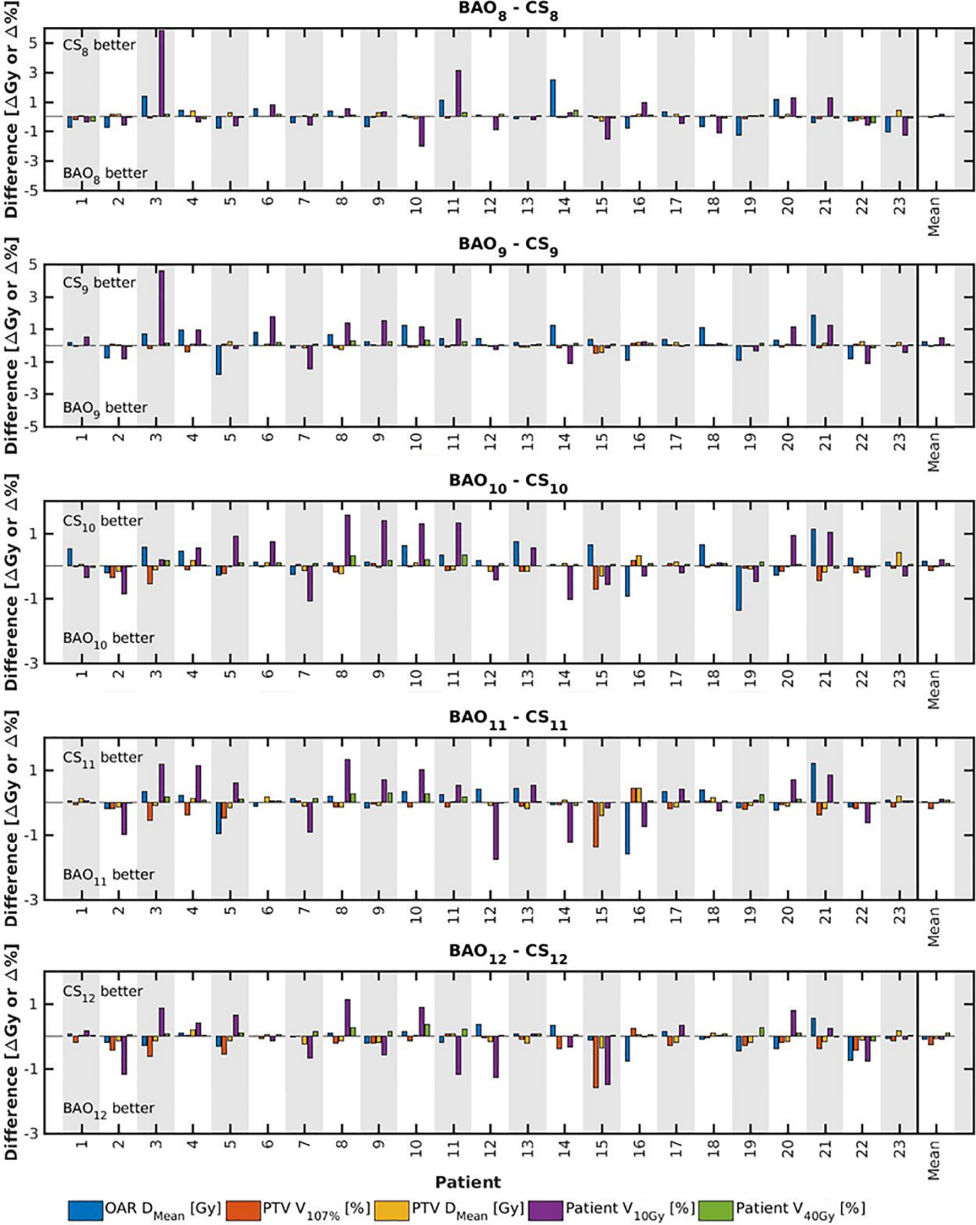
For all patients separately, plan parameter differences between CS_x_ and BAO_x_ for x=8-12 (upper panel) and CS_9_ (lower panel). The last columns show population mean differences. Leave-one-out data was used for CS_x_.

**Figure 4:**
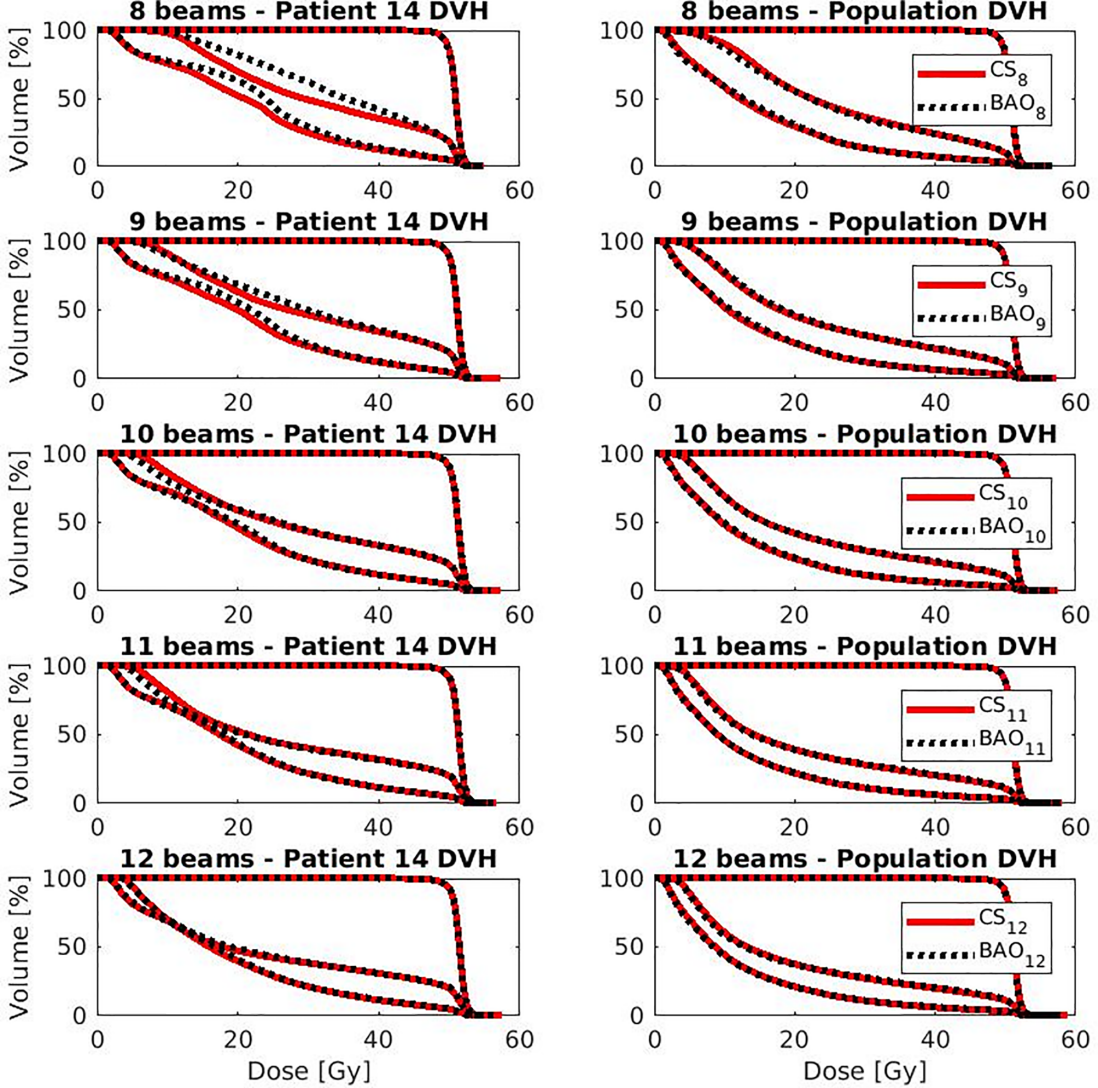
OAR and PTV DVHs for the patient with largest difference (i.e., patient 14 from Figure 4) (left column) and population average DVH (right column) for x=8-12. Leave-one-out data was used for CS_x_.

**Figure 5:**
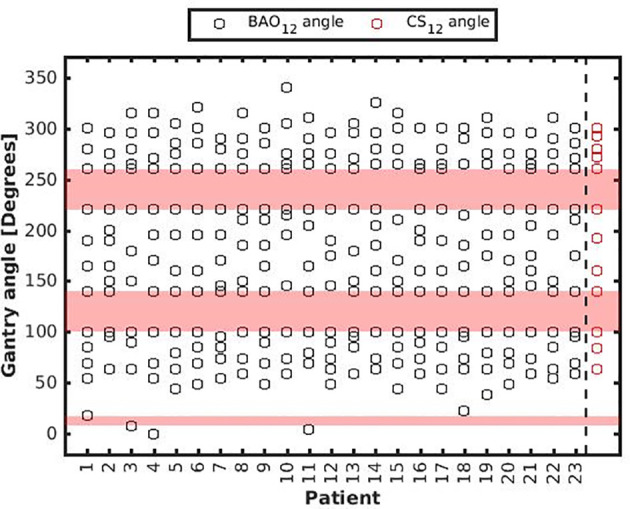
Patient-specific BAO_12_ beam angles. As a reference, CS_12_ beam angles were added with red markers in the last column, see also **Table 1**. BAAs are depicted in pink.

**Figure 6:**
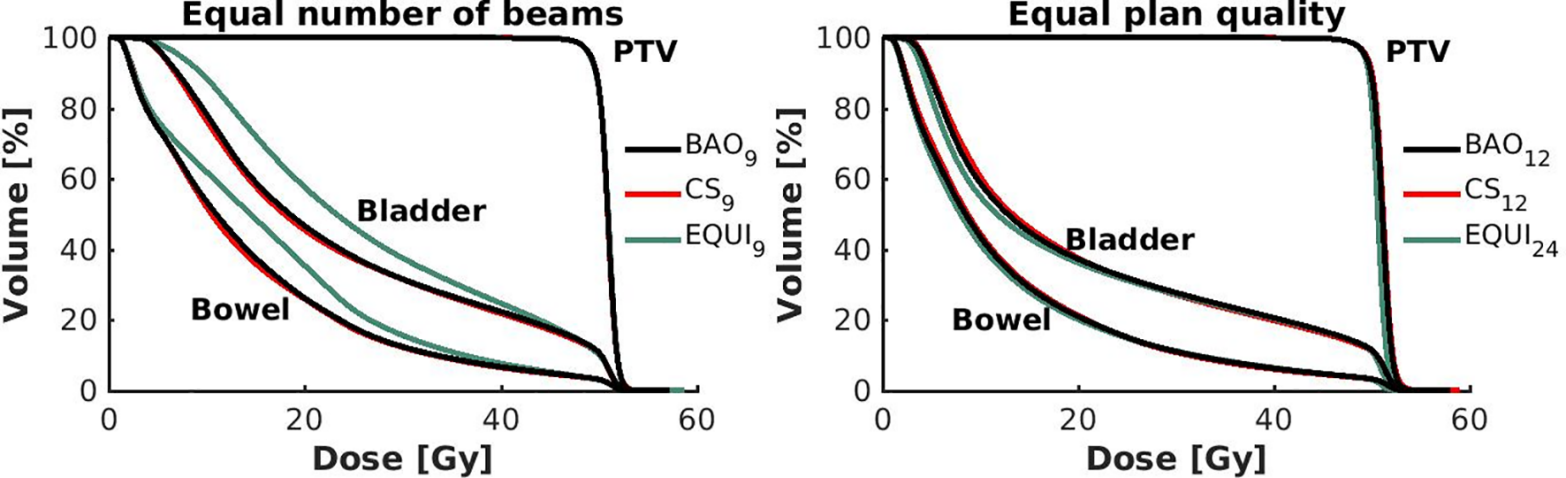
Left: Population mean DVHs for equal numbers of beams, showing higher bowel and bladder doses for equi-angular plans, while BAO and CS almost overlap. Right: Population mean DVHs for similar plan quality, using more beams for equi-angular set-ups (24 instead of 12).

**Figure 7:**
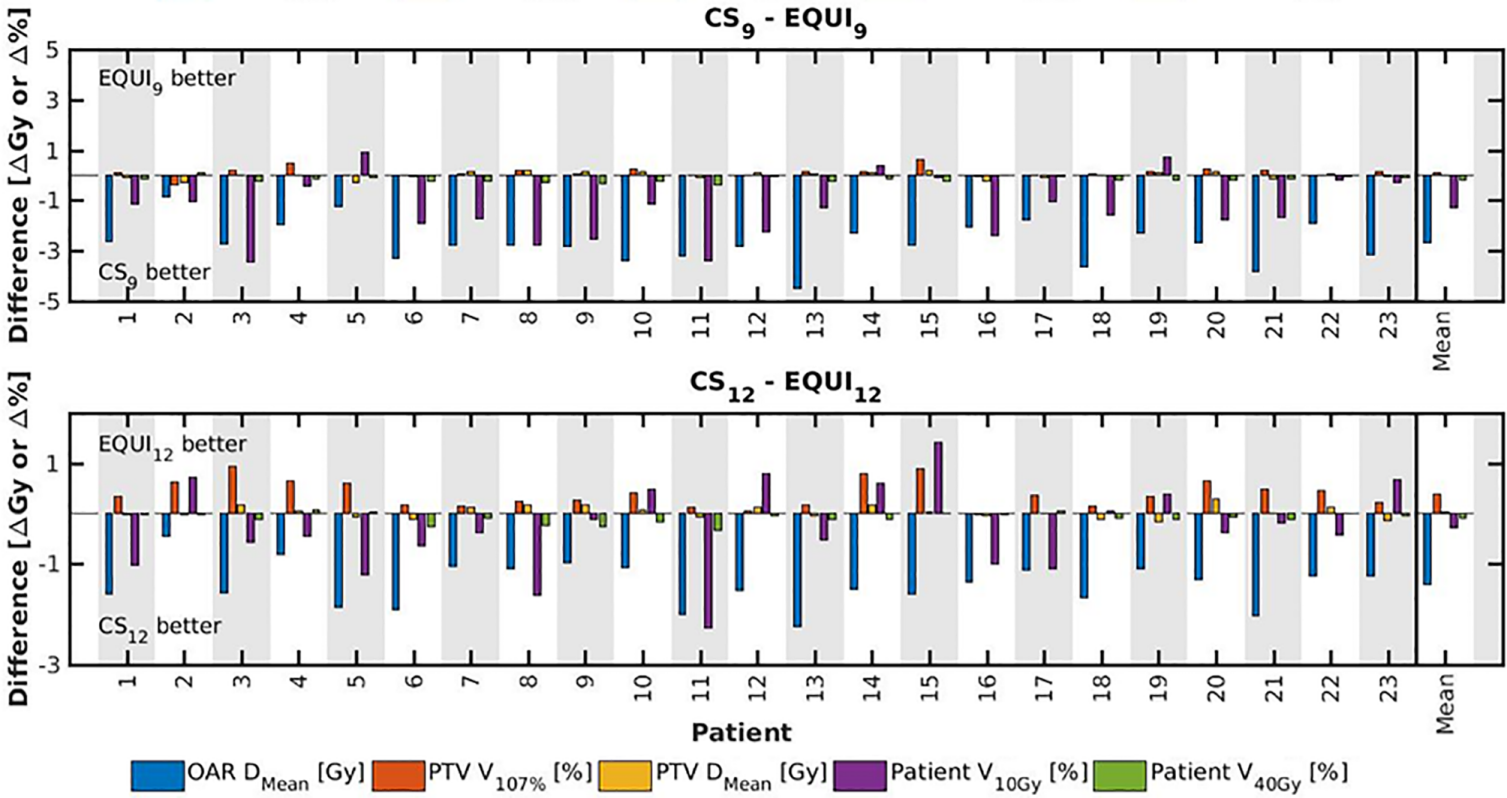
For all patients separately, plan parameter differences between CS_x_ and EQUI_x_ for x=9 and x=12. The las columns show population mean differences.

Several generated CS_7_ plans had PTV coverages as low as 90%, requiring major re-scaling to arrive at the desired 99% coverage (see *Materials and Methods* section), which then sometimes resulted in too large hot spots in the PTV (See *Discussion*). When excluding all 23 CS_7_ plans, the mean PTV coverage for the remaining 414 CS_x≥8_, BAO_x≥7_ and EQUI_x≥7_ plans, prior to rescaling, was 99.4%, range [98.6%-99.8%], i.e. the applied rescaling was minor. Mean dosimetric parameters of rescaled plans are compared in **Figure 2**, showing for CS_x_ plans mean values for left-out patients. P-values are presented in **Figures A1-A12** in Electronic Supplement A. **Figure 2** clearly illustrates the above-mentioned problems with CS_7_, and led to the conclusion that the proposed CS_x_ construction method did not properly work for x=7. For x≥8, high similarity between CS_x_ and BAO_x_ plans was observed in **Figure 3** and the population based DVHs in **Figure 4** confirm the high quality of the CS_x_ plans for all individual patients. **Figure 4** also shows the DVHs of patient 14, the patient with largest differences between CS_x_ and BAO_x_. Even for this patient the differences were limited. The data presented in **Figures 2**–**4** demonstrate generalizability for ≥8 beams. The final CS_x_, generated based on all 23 patients are presented in **Table 1**. **Figure 5** compares for the 23 study patients, patient-specific beam angle configurations in BAO_12_ plans with CS_12._ The final CS_x_ were used to generate data for **Figures 6** and **7** below.

**Table 1:** Beam angle configurations of the different CS_x_ based on all 23 patients.

	Beam Angles
CS_8_	64 84 100 140 192 220 260 292
CS_9_	64 84 100 140 160 192 220 260 292
CS_10_	64 84 100 140 160 192 220 260 292 300
CS_11_	64 84 100 140 160 192 220 260 272 292 300
CS_12_	64 84 100 140 160 192 220 260 272 280 292 300

### Evaluation and Comparison of BAO, CS and EQUI Plans

Some interesting observations were made:

- For the higher priority healthy tissues (OAR, bladder and bowel), dose reductions in BAO_x_, CS_x_ and EQUI_x_ plans with increasing x were steep. E.g. when moving from CS_8_ to CS_12_, only 4 beams more, the OAR D_Mean_ reduced from 16.2 Gy to 12.6 Gy, a 22% reduction (upper panels **Figure 2**).
- For fixed x, reductions in OAR doses in BAO_x_ and CS_x_ plans compared to EQUI_x_ where potentially meaningful, while dose delivery to the PTV (middle panels **Figure 2**) and patient (lower panels **Figure 2**) were similar. E.g. when using CS_12_ instead of EQUI_12_, OAR D_Mean_ reduced on average from 12.6 Gy to 14.1 Gy (11% reduction). These observations are confirmed by the population mean DVHs presented in **Figure 6 left** and the patient-specific plan comparisons for x=9 and x=12 in **Figure 7**.
- Increased dose delivery to the composite OAR, bowel and bladder in equi-angular plans could be compensated for by using more beams (upper panels **Figure 2**). This observation is supported by the population mean DVHs presented in **Figure 6 right**, showing that 24 equi-angular beams were needed to approach the quality of 12-beam class solution plans and 12-beam plans generated with BAO.

When using beam angle class solutions for plan generation instead of patient-specific beam angle optimization, calculation times were largely reduced. E.g. for CS_12_ plans, calculation times were 1-2 hours, which was a factor of 10 to 15 higher for BAO_12_ plans. Main time reduction is attributed to the fact that beam angle selection is no longer needed for the CS_12_ plans.

### Irradiation Through BAAs

The plans presented in this study were generated with full avoidance of the BAAs (**Figure 1**). Although not clinically applied at NKI, using beams going through the left- and right-inferior avoidance areas (**Figure 1**) is technically possible. The question rises to what extent allowing beams to pass through those BAAs might further improve quality of MRL plans. In Electronic Supplement B, computer-optimized patient-specific BAO was used to investigate the impact of allowing also beams pass through the left- and right-inferior avoidance areas, showing only minor plan quality improvements.

## Discussion

Current MRL systems require for each patient a selection of discrete angles of incidence for applied coplanar IMRT beams. The main aim of this paper was to explore the use of an advanced algorithm for integrated patient-specific beam angle and profile optimization to investigate computer-aided development of beam angle class solutions (CS) for avoiding the need of computationally intensive, patient-specific beam angle optimization (BAO), while maintaining the same high plan quality. Rectal cancer at a Unity system was used as a model case. Basically, constructed CS_x_ (x = number of included beam directions) contained most frequently occurring beam angles in BAO_12_ plans of a group of patients used for model construction. A leave-one-out validation approach demonstrated that the proposed construction methodology worked well for CS_x>7_ (i.e. resulting in a plan quality highly similar to BAO_x_), but not for x=7 (see below). CS_x_ and BAO_x_ plans for x=8-12 were compared to plans with equi-angular setups (EQUI) with up to 56 beams. All plans were fully automatically generated, allowing analyses based on a large number of treatment plans (437), and comparison of treatment approaches without well-known limitations of manual planning (26, 27).

While for x>7, quality of CS_x_ plans was highly comparable to BAO_x_, computation times dramatically reduced (for x=12: from 10-30 hours to 1-2 hours, depending on the patient). This renders planning with a CS favorable for generation of initial treatment plans in the treatment preparation phase, but it can also have consequences for daily adaptive re-planning at an MRL; when using a CS, daily re-optimization of beam angles (which would anyway be infeasible because of calculation times), is not needed as it will not result in plan improvements (as the CS work for all patients, they also work for different anatomies of the same patient). In this study, we did not investigate whether also beam arrangements established with individualized BAO prior to treatment would be robust for day-to-day anatomical variations during the fractionated treatments. This could be studied by comparing adaptive planning with and without BAO on repeat images.

The simpler and more consistent workflow with a CS may also be favorable for QA, both pre-treatment and after plan adaptation.

For all x>7, BAO_x_ and CS_x_ plans clearly outperformed EQUI_x_ plans regarding quality, especially for doses delivered to the bladder and bowel. Equal quality of BAO/CS and EQUI plans could be obtained by enhancing the number of beams in the EQUI plans. E.g. the quality of EQUI plans with 24 beams was similar to BAO and CS plans with 12 beams. However, the use of substantially enhanced numbers of beams would increase plan optimization times, which is unfavorable, especially in a setting of daily adaptive re-planning. It would also result in more complex QA. There may also be clinical reasons for avoiding multi-beam treatments, e.g. for children and lung tumors. Overall, EQUI plans with many beams (up to 56) had the highest quality. This observation could possibly hint at a superior plan quality if VMAT would be implemented for the investigated MRLs, although performance of VMAT could possibly be lower than IMRT with many-beam EQUI setups due to delivery limitations with VMAT. The Unity MRL has a fast rotating gantry (6 rpm). This could possibly yield fast delivery of multi-beam equi-angular setups feasible, which would amongst other parameters depend on gantry deceleration and acceleration times. To our knowledge, no peer-reviewed publications on the topic have appeared so far.

For CS_7_ we observed too high PTV V_107%_. This is a direct effect of the large amount of rescaling required to obtain the requested PTV coverage. This showed that the proposed CS_x_ construction methodology is less suited for low x. Most likely, beam angle selection is more sensitive if few beams are involved.

In this study, also the generation of CS_x<12_ was based on BAO_12_ plans. From experience we know that the sequential BAO in the applied optimizer (7) will not always be fully optimal for lower beam numbers. As obtaining the highest possible plan quality with the CSs was the aim in this study, we avoided using BAO_x_ plans with small x for CS generation, and always used BAO_12_ plans instead.

The analyses presented in Electronic Supplement B demonstrate that the impact of respecting left-and right-inferior BAAs on plan quality is small: addition of one extra beam has a much larger impact on plan quality than keeping the beam number fixed, but allowing beams to pass through BAAs. This could of course be different for other tumor sites.

Although this work was done for the Unity MRL, we believe it is also relevant for the MRIdian^®^ system (Viewray, Oakwood Village, Ohio, USA). Also for the MRIdian^®^, dose is delivered with step-and-shoot IMRT, requiring selection of discrete beam angles. The system has a gantry rotation speed of 0.5 rpm, and also for this system, gantry deceleration and acceleration times will contribute to delivery times of multi-beam treatments compared to treatment with fewer (well-selected) beams. As for any system, large increases in beam numbers would result in enhanced computation times for daily adaptive re-planning, and there would be an impact on QA.

Advanced options for beam angle optimization are currently lacking in commercial TPSs. Many studies have demonstrated the benefit of such algorithms for non-coplanar treatment (5–11, 28). This study demonstrates that such algorithms could also enhance treatment plan quality for coplanar MRL treatments. Although treatment planning based on beam angle CSs could avoid patient-specific BAO, the CSs proposed in this study were developed and validated with BAO plans. In the absence of advanced BAO functionality, many centers work with beam angle CSs derived with manual trial-and-error planning. The NKI decided to replace their original 9-beam CS, obtained from manual planning, with the CS_9_ developed in this study. Lack of advanced BAO in the commercial TPSs for MRL could complicate demonstration of added value of these systems in clinical studies. Till implementation of BAO tools in these systems, institutes with advanced in-house tools could develop CSs, which could then be used in (multi-center) clinical studies.

The CSs in this study were developed for one specific treatment planning protocol for rectal cancer patients. The validity for other protocols was not investigated and will be subject of further research. More studies for other tumor sites are also warranted to explore what beam numbers and beam angle configurations are needed for high-quality plans, and to investigate required numbers of model patients.

The RATING guidelines for treatment planning studies (29) assisted in preparing the manuscript. Two investigators (RB, LR) independently filled out the Rating score list, arriving at scores of 80% and 83%.

## Conclusion

For rectal cancer patients treated at a Unity MRL, computer-generated beam angle CS could replace individualized BAO without loss in plan quality, while reducing planning complexity and calculation times, and resulting in a simpler clinical workflow. Both CS and BAO treatments largely outperformed multi-beam equi-angular treatment. With the developed high-quality CS, time consuming beam angle re-optimization in daily adaptive MRL treatment could be avoided, as it would not enhance plan quality. Further research on computerized development of beam angle class solutions for MRL treatment planning is warranted. There is a need for implementation of advanced beam angle optimization tools in TPSs of MRL systems.

## Data Availability Statement

All relevant data are within the paper and its supplementary files. Access to raw-data underlying the findings in this paper will be made possible on request to corresponding author.

## Ethics Statement

Ethical review and approval was not required for the study on human participants in accordance with the local legislation and institutional requirements. Written informed consent for participation was not required for this study in accordance with the national legislation and the institutional requirements.

## Author Contributions

Conceptualization, BH and LR. Methodology, RB, LR, and BH. Software, RB and SB. Data gathering and curation RB and LR. Data analysis RB and LR. Data interpretation: all. Writing—original draft preparation, RB and LR. Writing—review and editing, RB, LR, BH, TJ, PR, BT, SB, and J-JS. Supervision, LR and BH. All authors contributed to the article and approved the submitted version.

## Funding

This work was in part funded by a research grant of Elekta AB (Stockholm, Sweden). Erasmus MC Cancer Institute also has a research collaboration with Accuray Inc, Sunnyvale, USA. The funders were not involved in the study design, collection, analysis, interpretation of data, the writing of this article or the decision to submit it for publication.

## Conflict of Interest

NKI is a member of the Elekta MR-Linac consortium.

The remaining authors declare that the research was conducted in the absence of any commercial or financial relationships that could be construed as a potential conflict of interest.

## Publisher’s Note

All claims expressed in this article are solely those of the authors and do not necessarily represent those of their affiliated organizations, or those of the publisher, the editors and the reviewers. Any product that may be evaluated in this article, or claim that may be made by its manufacturer, is not guaranteed or endorsed by the publisher.
