# Supplementary material for: MR-Linac Radiotherapy – The Beam Angle Selection Problem
Source: Front Oncol. 2021 Oct 1;11:717681. doi: 10.3389/fonc.2021.717681 (PMC8518312; doi:10.3389/fonc.2021.717681)
Supplement: Supplementary file 1 [file DataSheet_1.docx]

**Electronic Supplement A**

**Figures A1-A12**. Mutual comparisons of all CS_x≥7_, BAO_x≥7_ and EQUI_x≥7_ beam configuration approaches for each of the evaluated dosimetric plan parameters of the autoplans. Above the diagonal, median differences for patients 1-23 are presented. Blue values indicate differences in favour of the treatment on the row, orange values indicate differences in favour of the treatment on the column. On the diagonal, absolute population mean plan parameter values for all beam configuration approaches are presented. Below the diagonal, p-values for the plan parameter differences, presented above the diagonal, are reported. NS = p>0.05, green values = p<0.05


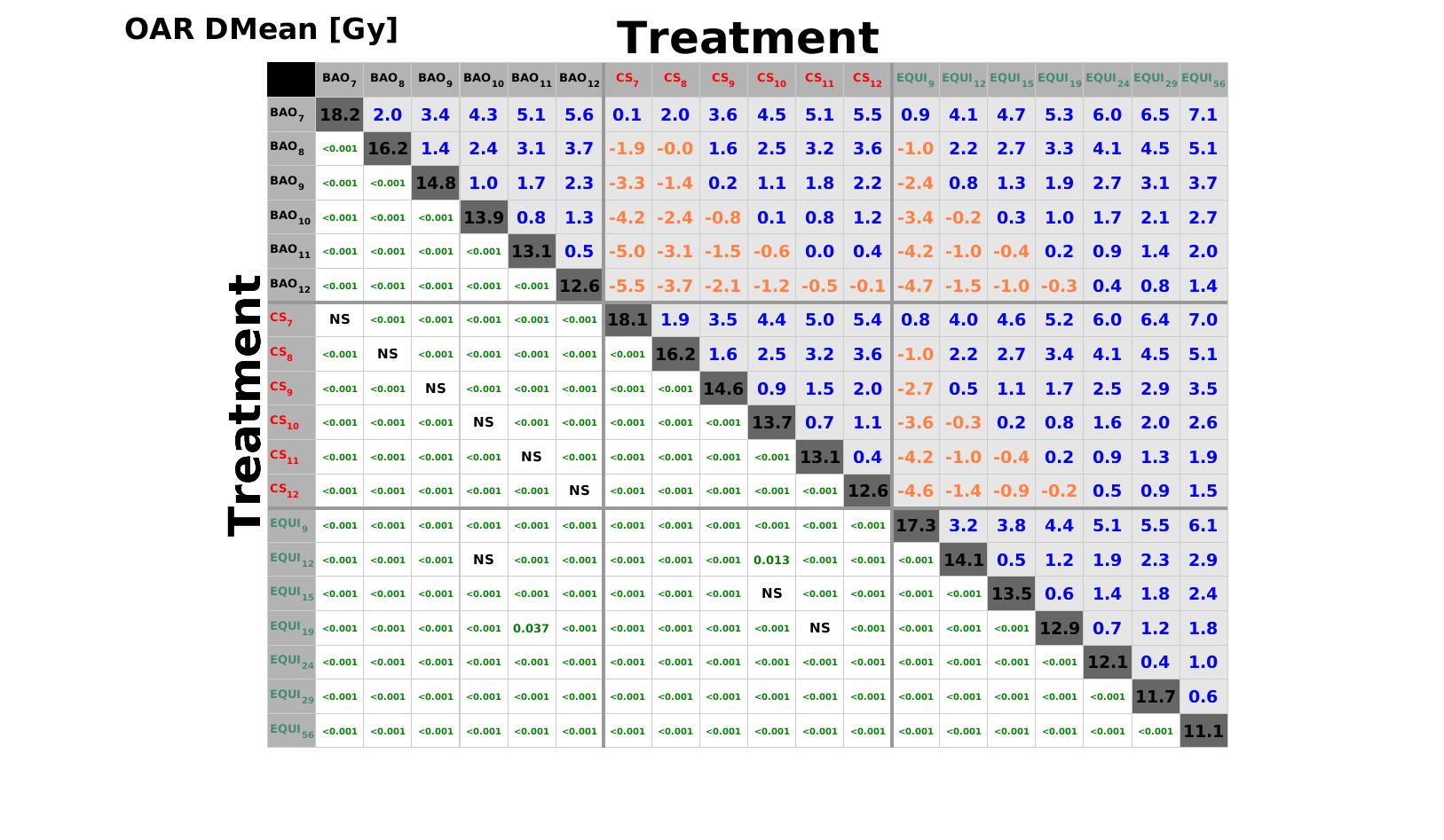


Figure A1: Mutual comparisons for OAR Dmean


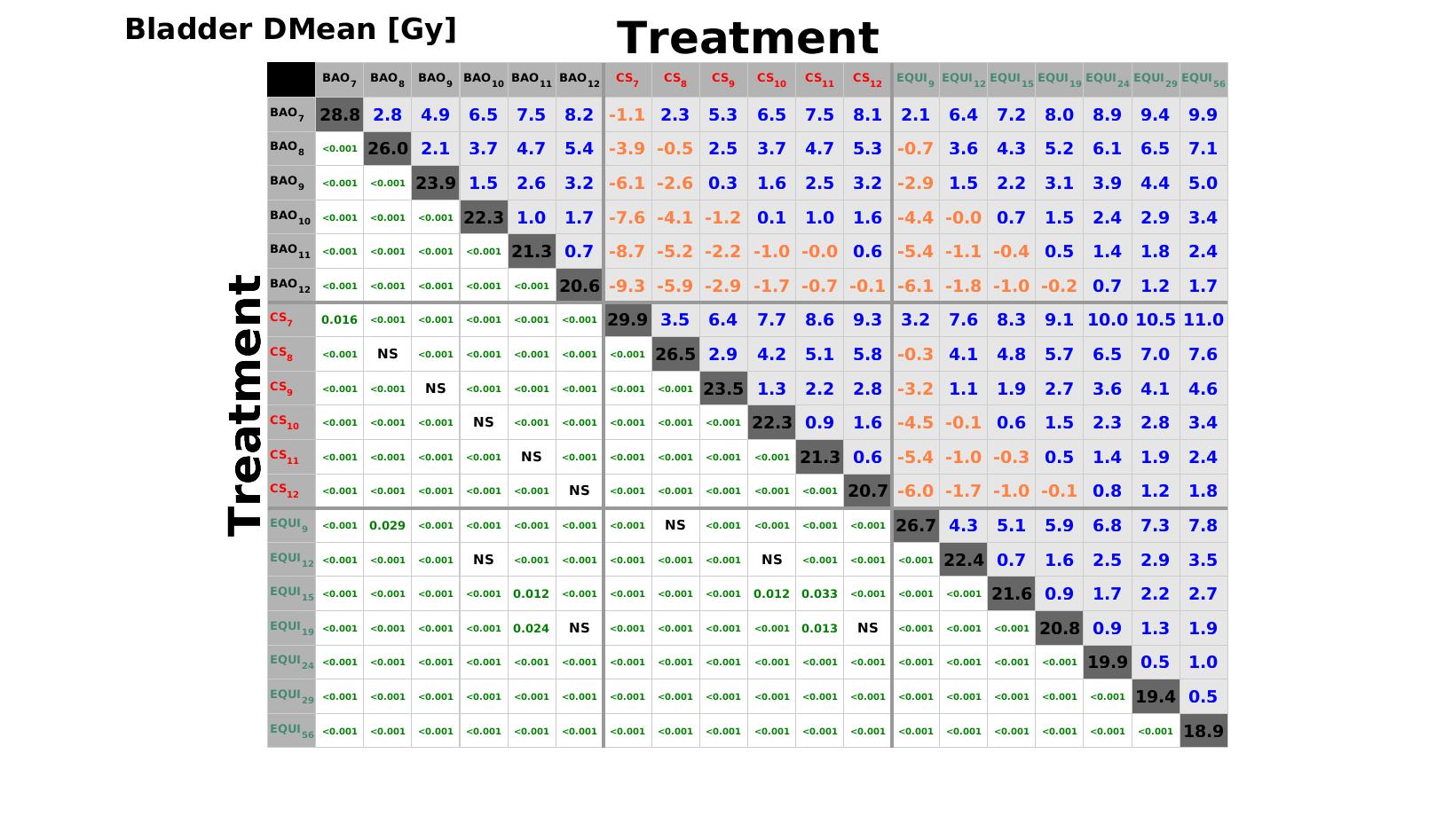


Figure A2: Mutual comparisons for bladder Dmean


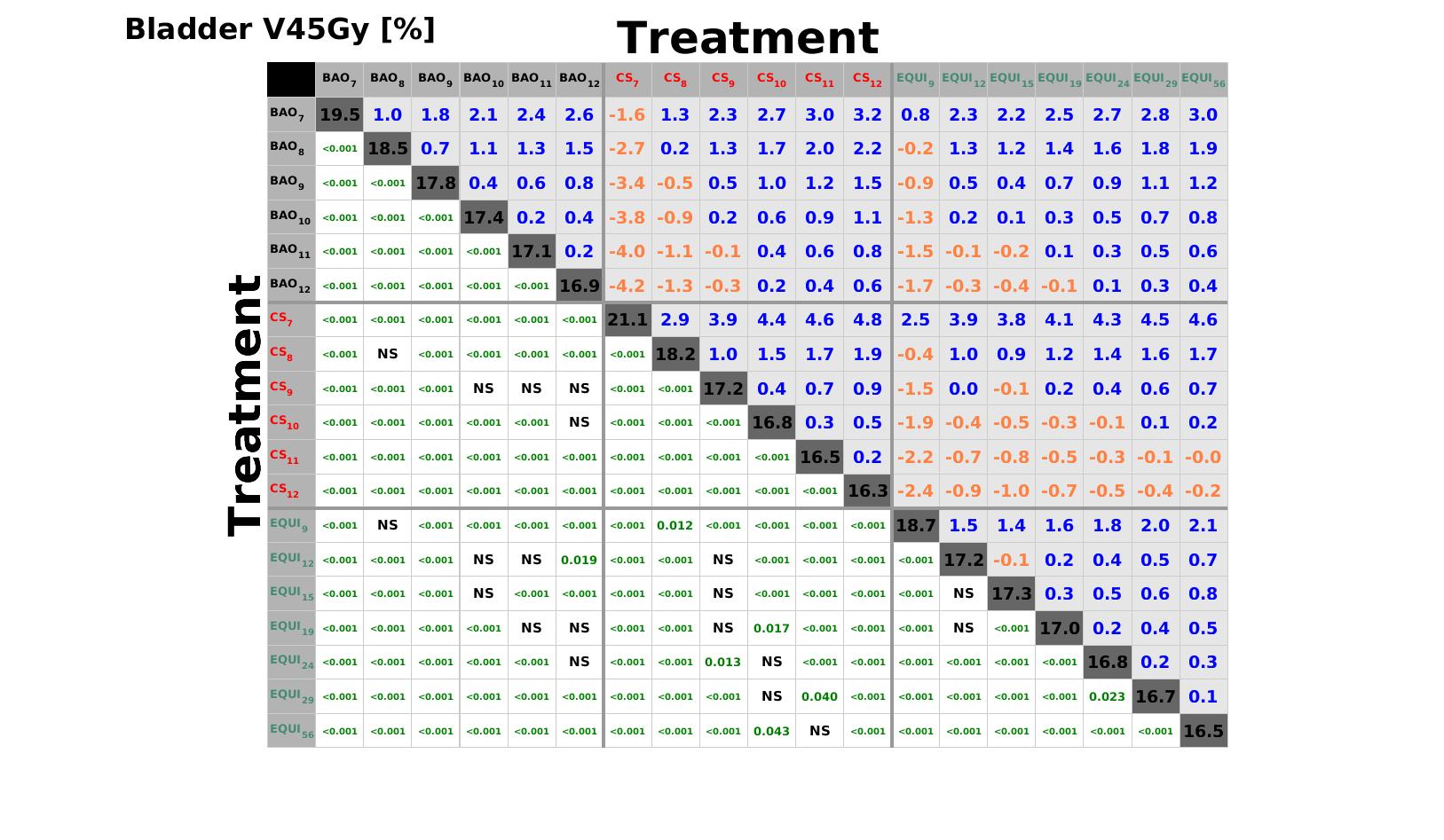


Figure A3: Mutual comparisons for bladder V_45Gy_


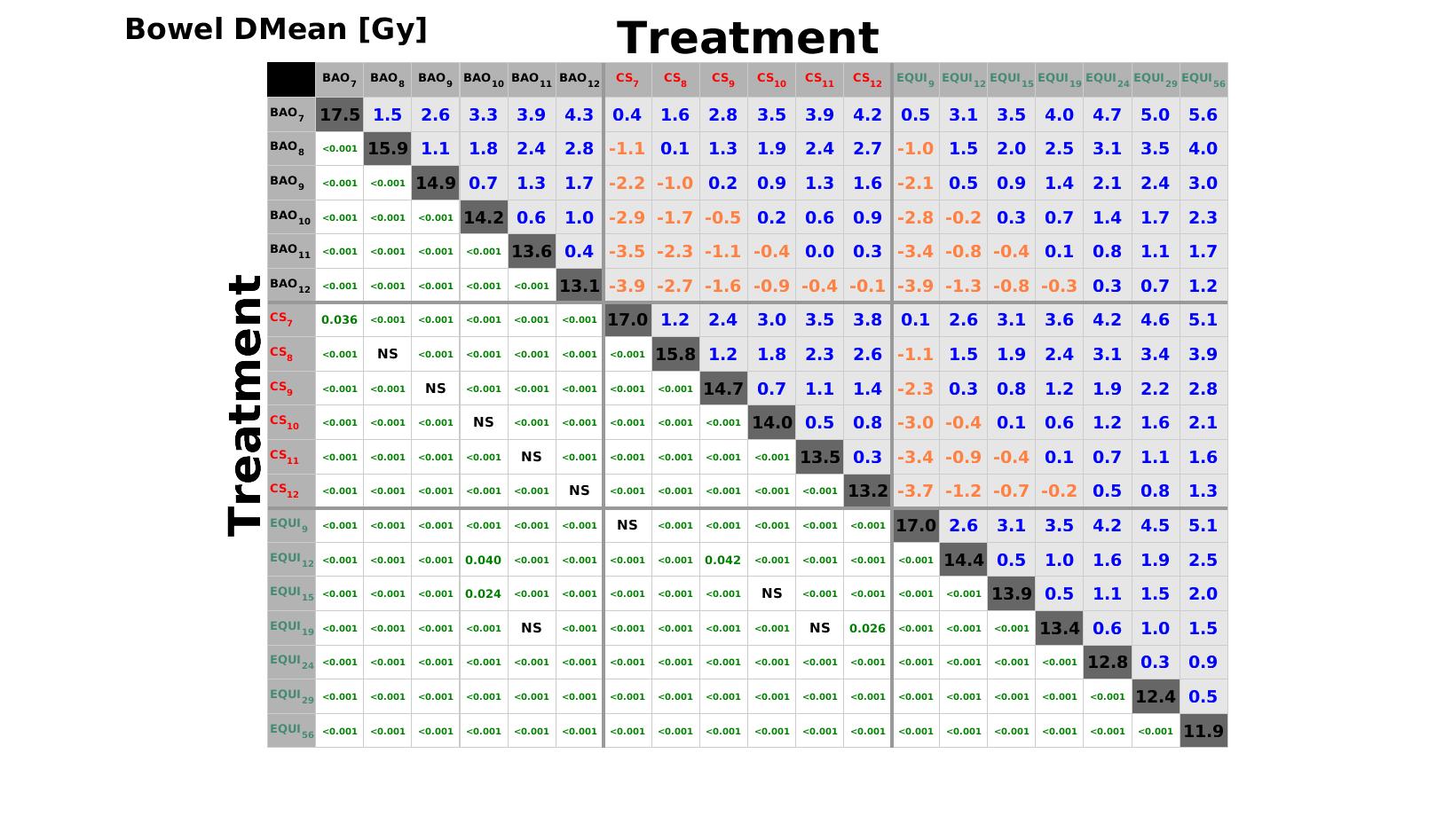


Figure A4: Mutual comparisons for bowel Dmean

*
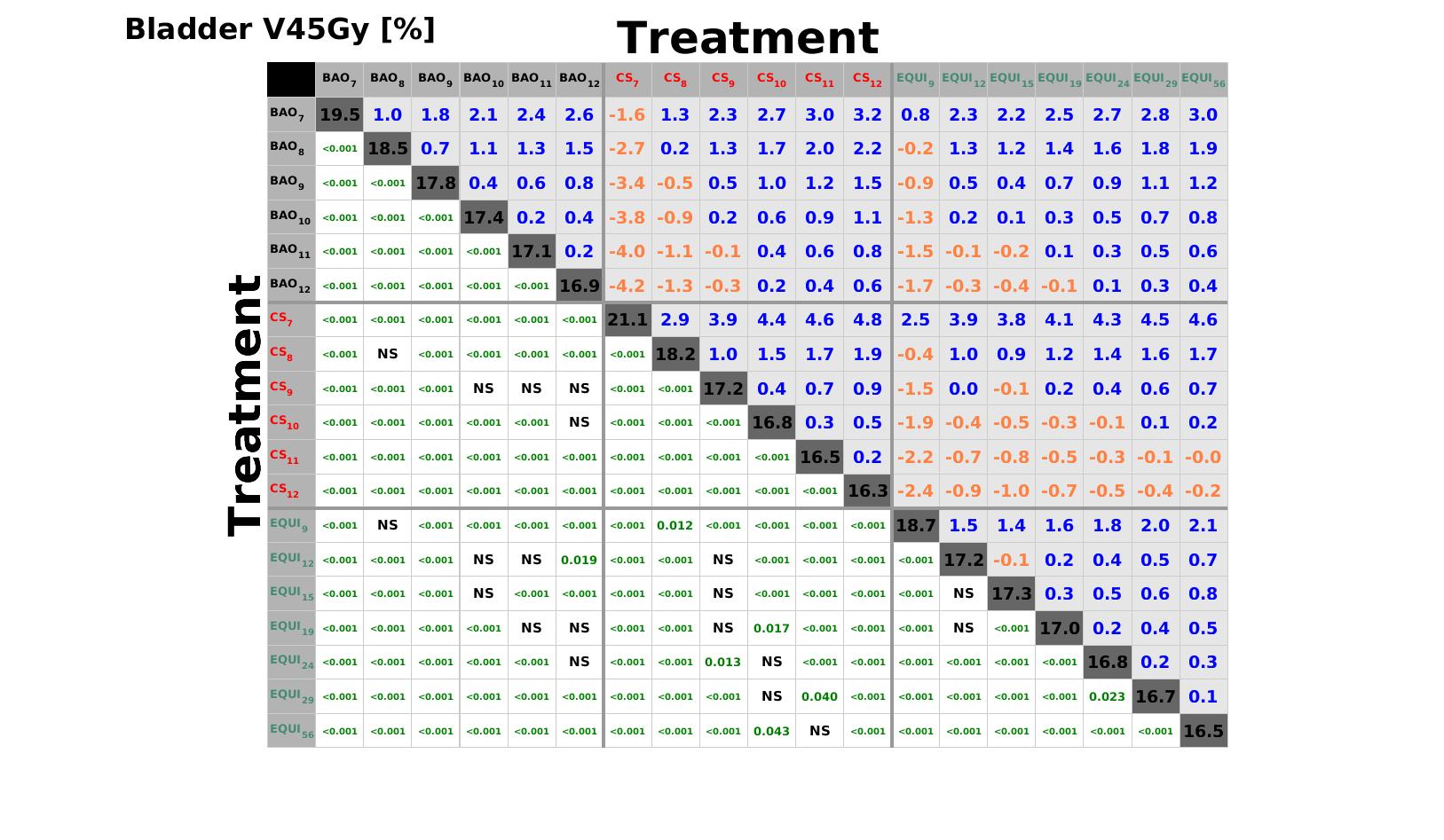
*

*Figure A5: Mutual comparisons for bowel V_45Gy_*

*
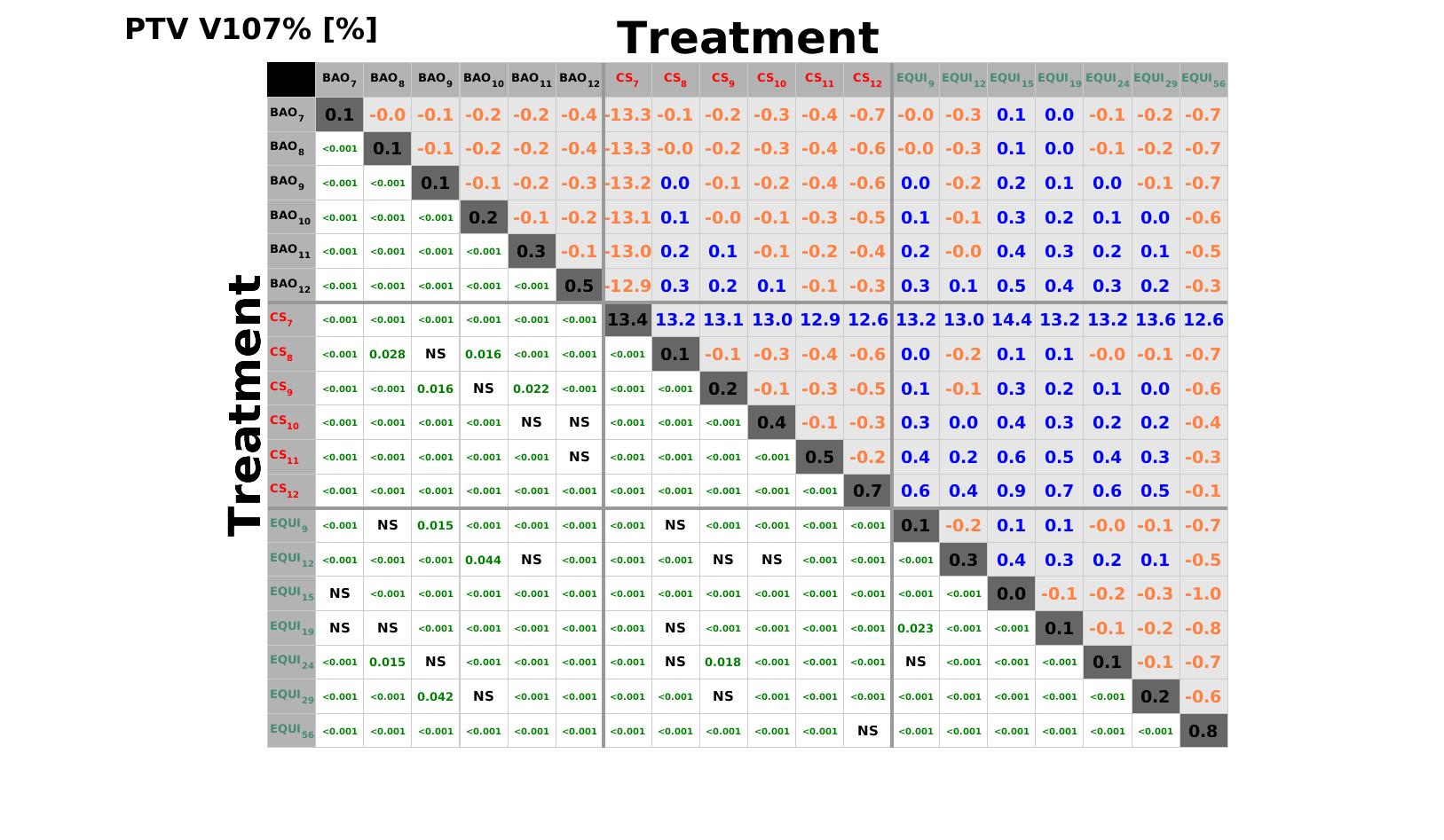
*

*Figure A6: Mutual comparisons for PTV V_107%_*

*
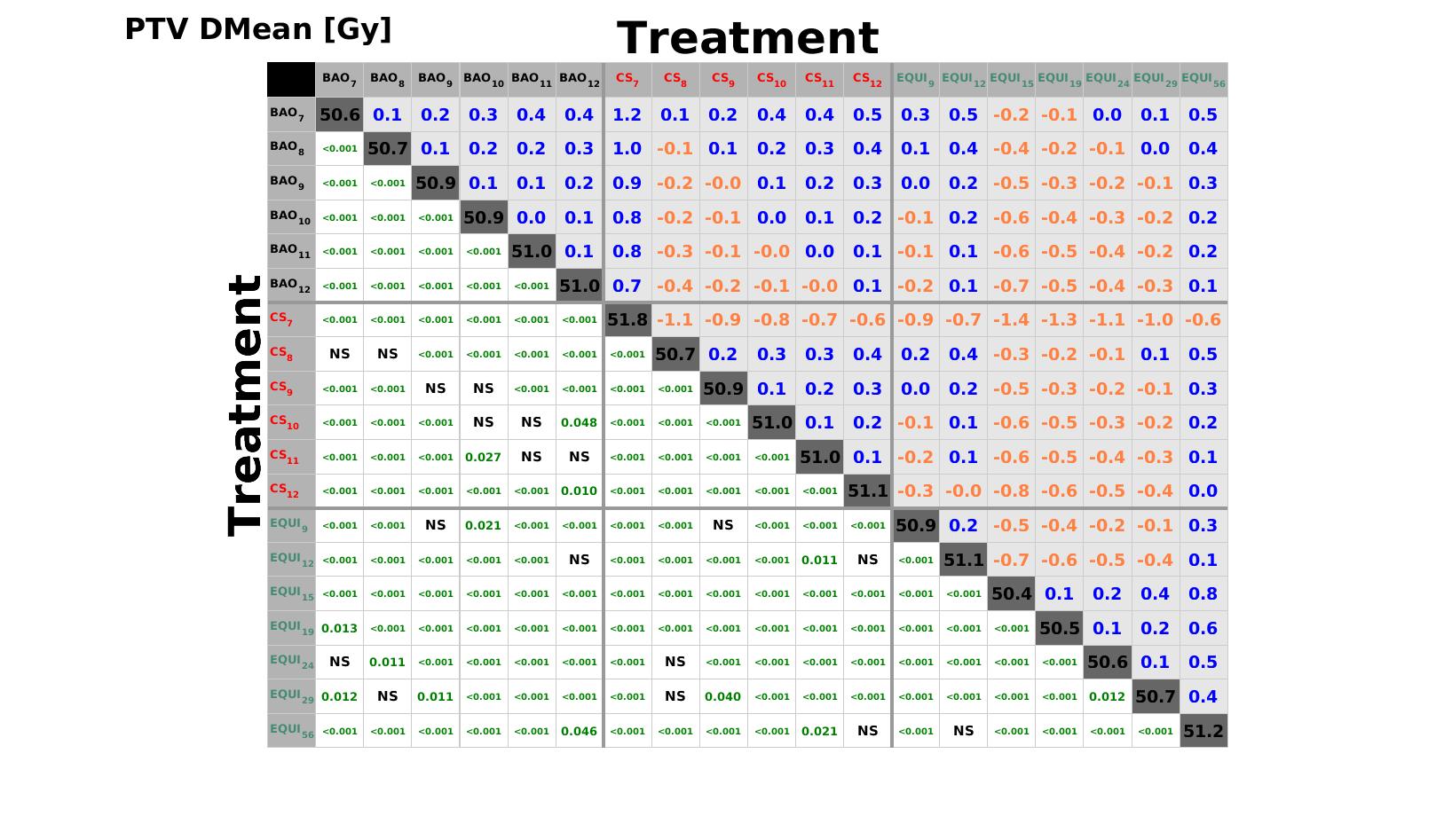
*

*Figure A7: Mutual comparisons for PTV Dmean*

*
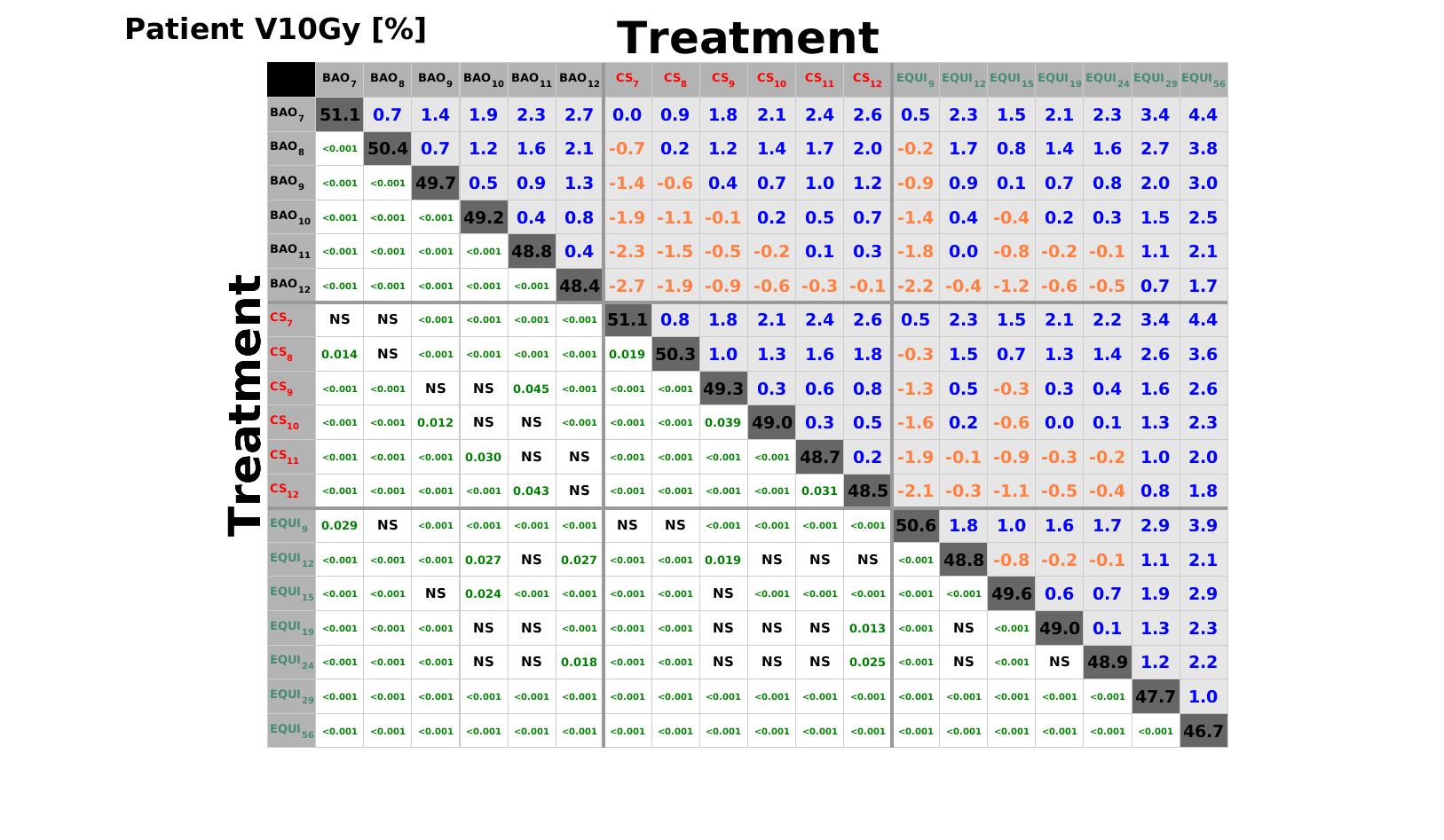
*

*Figure A8: Mutual comparisons for patient V_10Gy_*

*
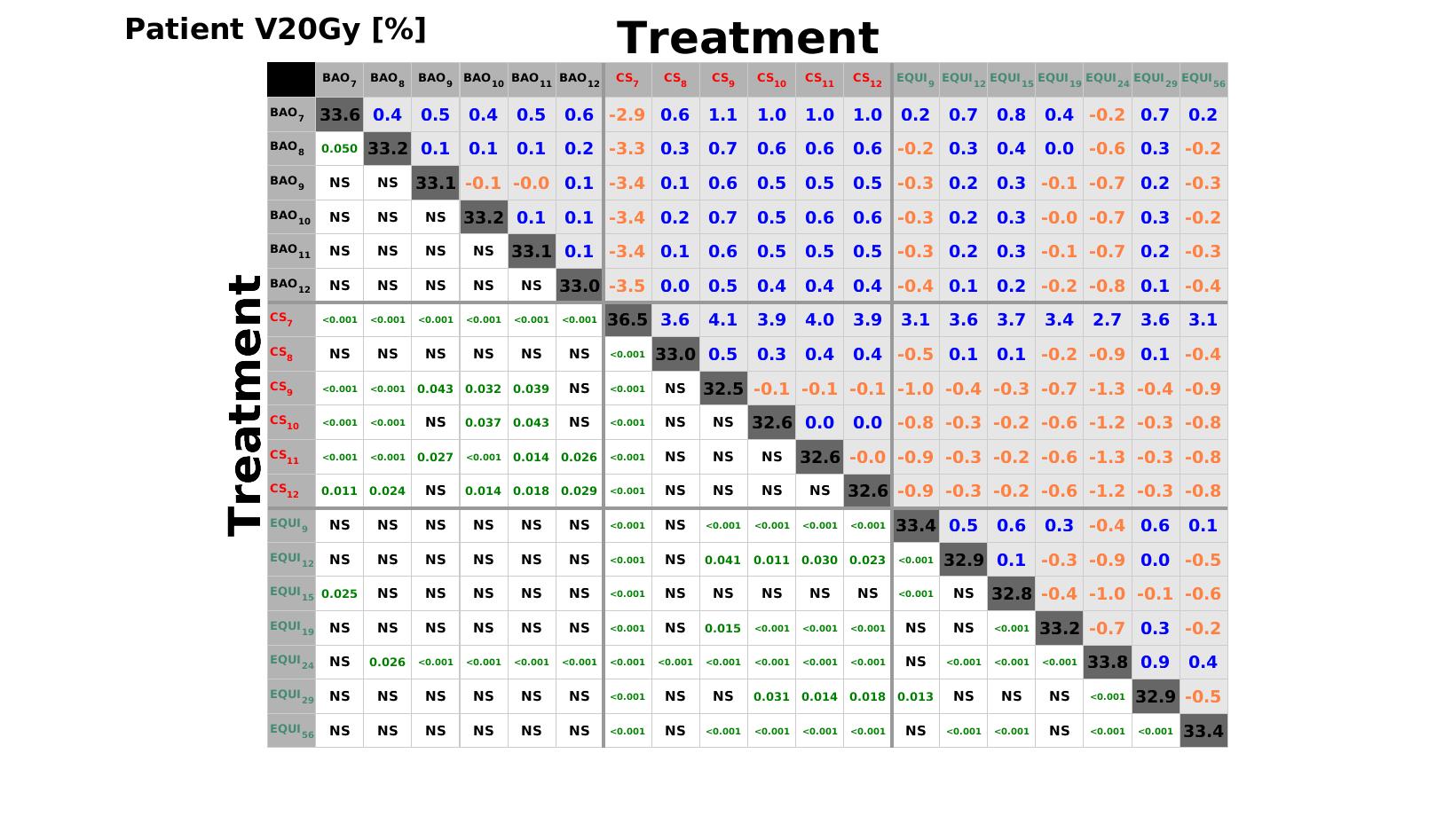
*

*Figure A9: Mutual comparisons for patient V_20Gy_*

*
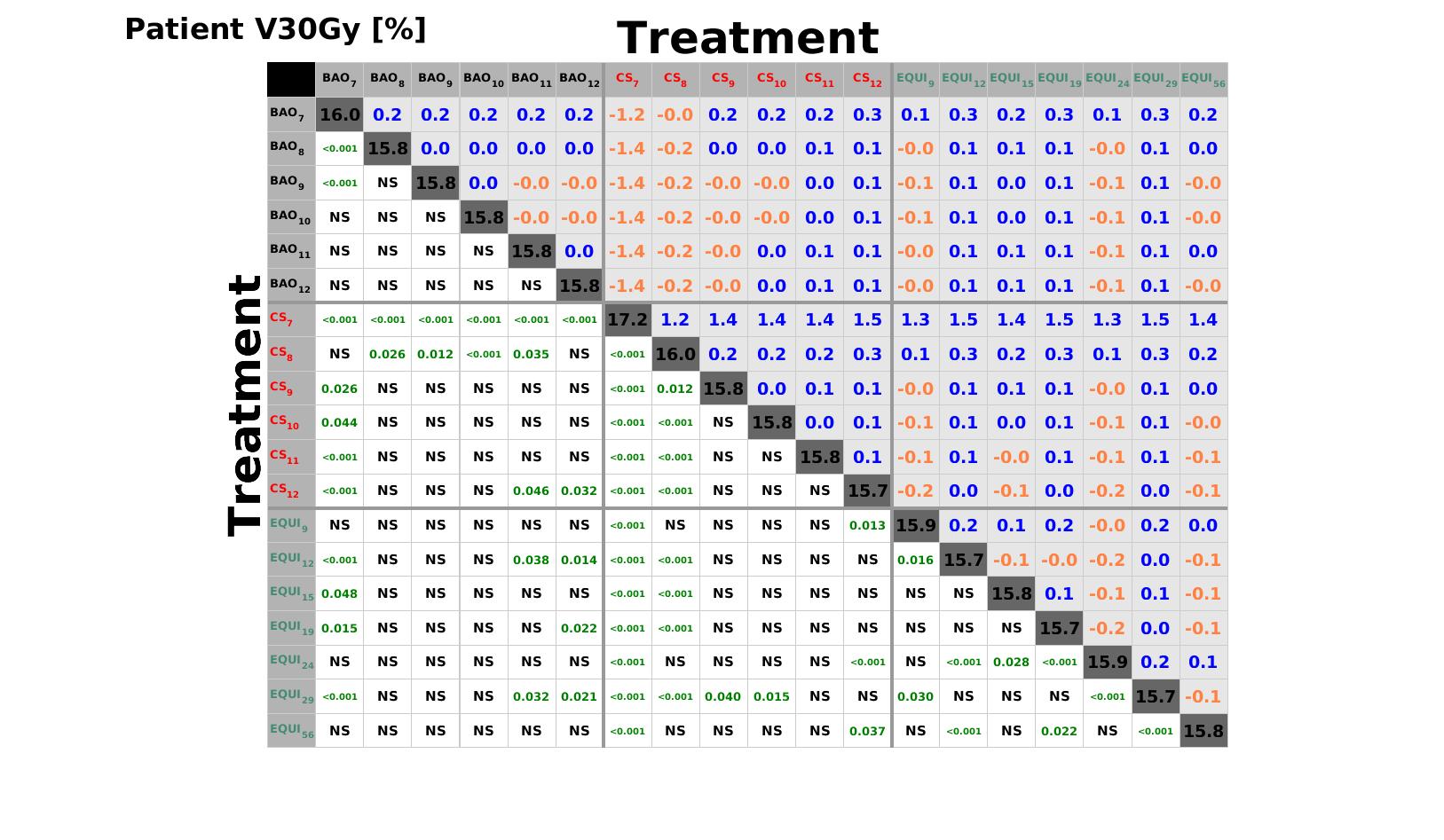
*

*Figure A10: Mutual comparisons for patient V_30Gy_*

*
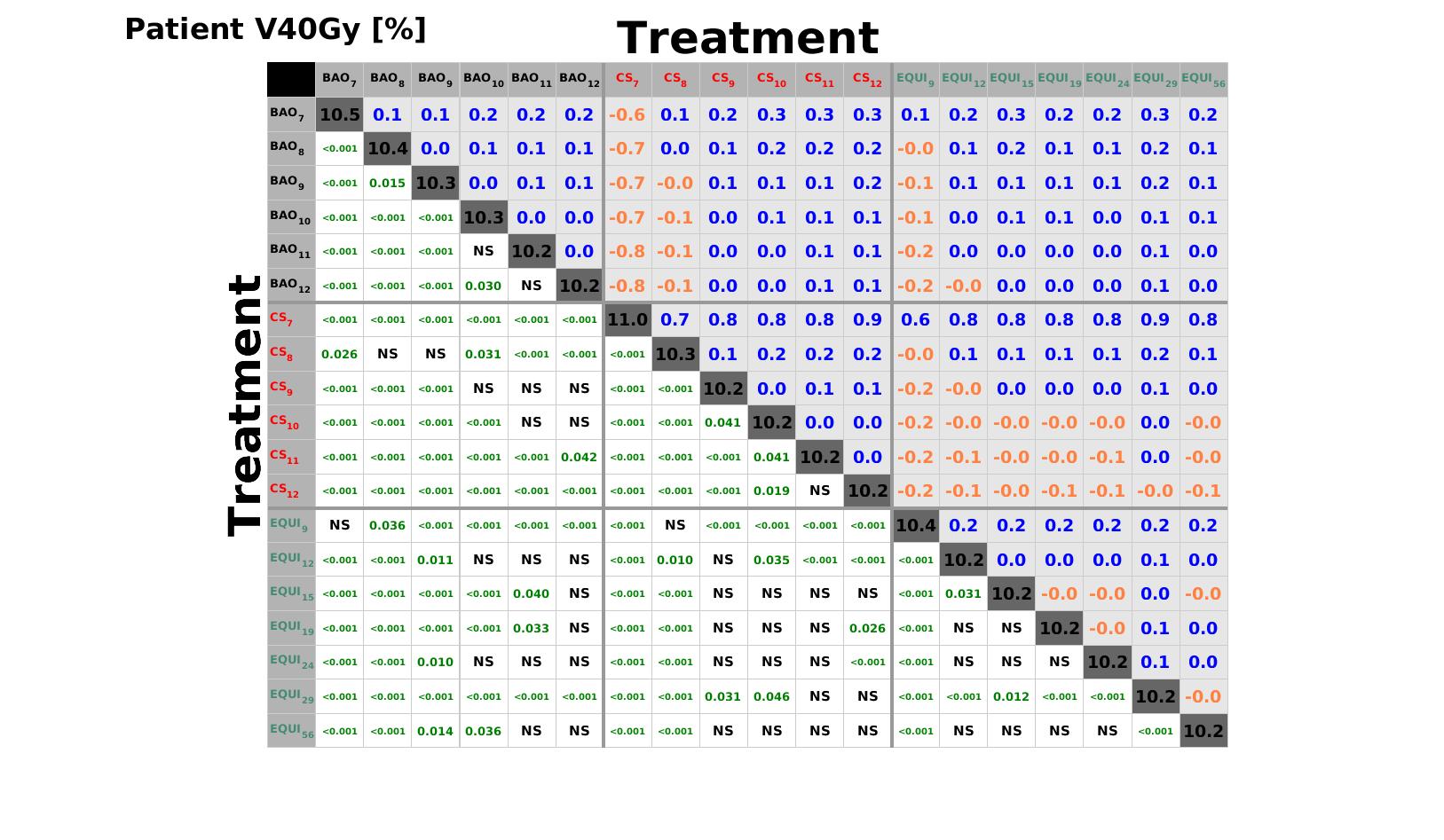
*

*Figure A11: Mutual comparisons for patient V_40Gy_*

*
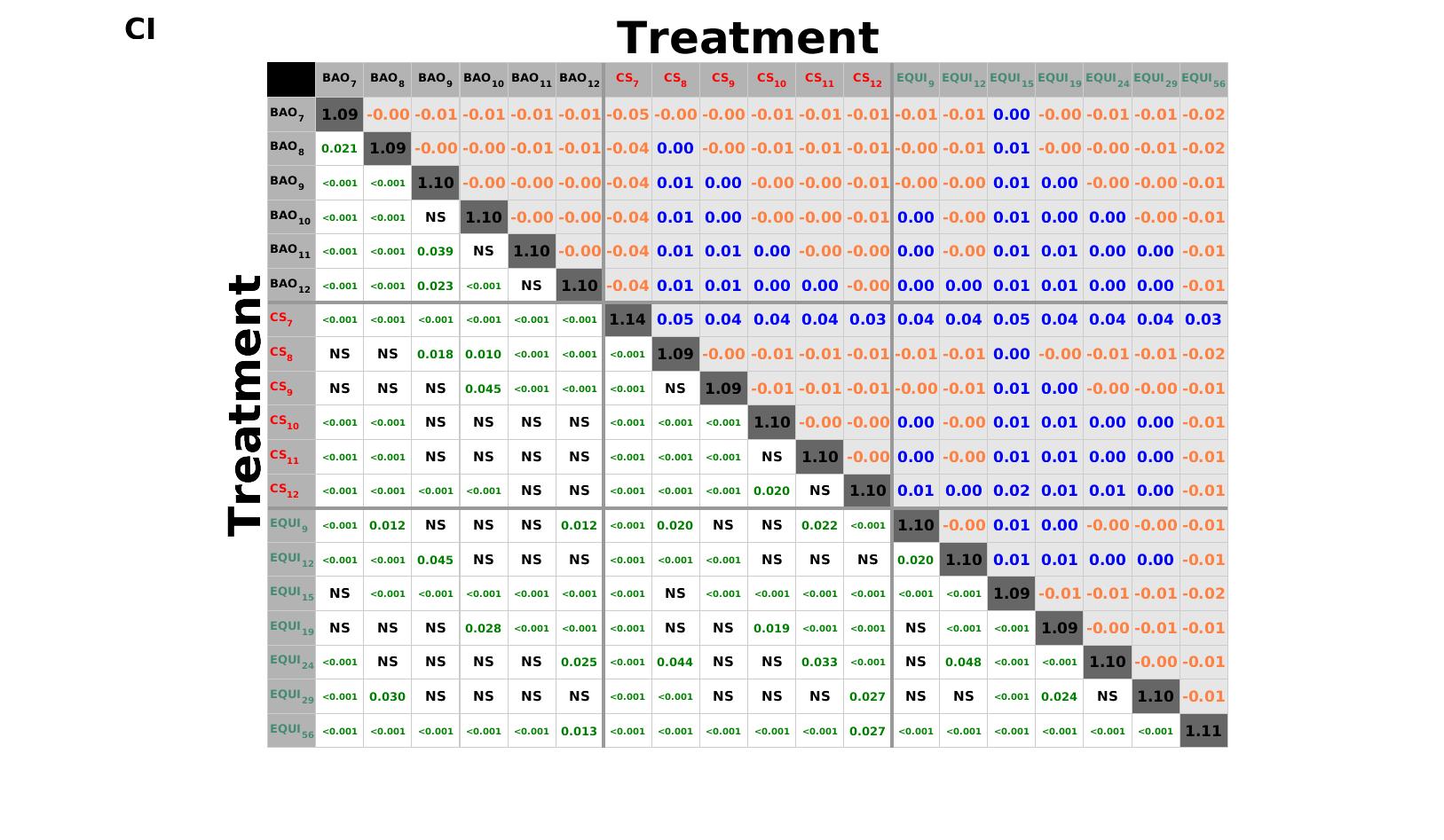
*

*Figure A12: Mutual comparisons for CI*
