# Supplementary material for: MR-Linac Radiotherapy – The Beam Angle Selection Problem
Source: Front Oncol. 2021 Oct 1;11:717681. doi: 10.3389/fonc.2021.717681 (PMC8518312; doi:10.3389/fonc.2021.717681)
Supplement: Supplementary file 2 [file DataSheet_2.docx]

**Electronic Supplement B**

*Impact of beam angle avoidance areas (BAAs) on plan quality*

Although not clinically applied at NKI, using beams going through the left- and right-inferior avoidance areas (pink inferior areas in Fig. 1) is technically possible. While it is possible to treat through the rails, the control system would not allow any treatment through the cryostat pipe. In this appendix, computer-optimized patient-specific BAO was used to investigate the impact of allowing also beams passing through the left- and right-inferior avoidance areas. To this purpose, BAO_9_ and BAO_12_ plans were generated for all 23 study patients, but now for a beam candidate space that included also these BAAs. BAO_9_ and BAO_12_ plans generated with and without these BAAs were then compared.


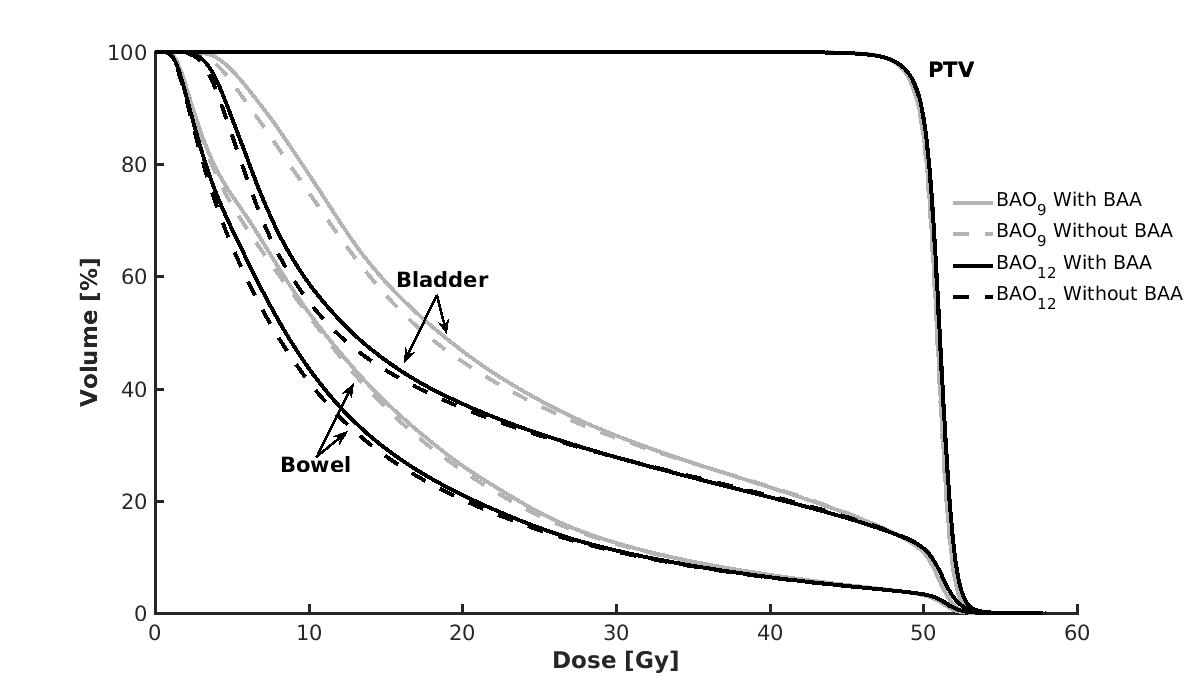


**Figure B1:** Population mean DVHs for BAO plans with 9 and 12 beams, generated with and without consideration of the left- and right-inferior beam avoidance areas (BAA) in the beam angle optimization.

Population mean DVHs for the OARs and PTV are presented in Fig. B1. For all patients, allowing dose delivery through the BAAs resulted in a median(range) selection of 2(1-5) and 4(3-7) beams out of 9 and 12 beams, going through these BAAs, respectively. Irradiating through BAAs led to small improvements in bowel and bladder DVHs in both 9 and 12 beam plans, resulting in median reductions in OAR D_Mean_ of 0.5 Gy (p = 0.005) and 0.1 Gy (p<0.001), respectively. The large distances between the mean OAR DVHs of 9 beam plans and 12 beam plans in Fig. B1 suggest that addition of one extra beam in plans that are generated while respecting BAAs has a much larger impact on plan quality than keeping the beam number fixed, but allowing beams to pass through BAAs.
